# Fabrication and Performance Evaluation of the Helmholtz Resonator Inspired Acoustic Absorber Using Various Materials

**DOI:** 10.3390/mi11110983

**Published:** 2020-10-31

**Authors:** Sung Ho Lee, Bong Su Kang, Gyu Man Kim, Yong Rae Roh, Moon Kyu Kwak

**Affiliations:** Department of Mechanical Engineering, Kyungpook National University, Daegu 41566, Korea; lee_sh@knu.ac.kr (S.H.L.); bong-su@knu.ac.kr (B.S.K.); gyuman.kim@knu.ac.kr (G.M.K.); yryong@knu.ac.kr (Y.R.R.)

**Keywords:** Helmholtz resonator, acoustic attenuation, photolithography, ultrasound, microscale

## Abstract

A soundwave is transmitted by adjacent molecules in the medium, and depending on the type of sound, it exhibits various characteristics such as frequency, sound pressure, etc. If the acoustic wavelength of the soundwave is sufficiently long compared with the size of an acoustic element, physical analysis within the sound element could be simplified regardless of the shape of the acoustic element: this is called “long wavelength approximation”. A Helmholtz resonator, a representative acoustic element which satisfies the “long wavelength theory”, consists of a neck part and a cavity part. The Helmholtz resonators can absorb certain frequencies of sound through resonance. To exhibit attenuation properties at ultrasound range, the Helmholtz resonator should be made into a microscale since Helmholtz resonators should satisfy the “long wavelength approximation”. In this study, Helmholtz resonator inspired acoustic elements were fabricated using MEMS technology, and acoustic attenuation experiments in a water bath were conducted using various shapes and materials. As a result, the fabricated samples showed admirable attenuation properties up to ~13 dB mm^−1^ at 1 MHz. The results were analyzed to derive the necessary conditions for the fabrication of acoustic elements with acoustic attenuation properties in ultrasound range.

## 1. Introduction

With the development of MEMS (Micro-Electro-Mechanical Systems) technology, micro/nano-structure fabrication technology has enabled the fabrication of complex shaped micro-structures. Much research into the development of various functional surfaces using micro/nano-structures has been actively conducted and widely applied in various fields such as biomimetics inspired by nature, lab on a chip, semiconductor manufacturing, optical field, and even acoustic field [1,2,3,4,5,6,7,8,9,10,11,12,13,14]. In addition, research for improving the productivity of the functional surface composed with micro/nano-structures has been actively carried out [15,16,17,18,19,20,21,22]. Recently, as mentioned before, in the acoustics field, many novel studies have been introduced by using microscale acoustic devices to utilize new characteristics by MEMS technology; generally, microscale acoustic elements have been covered in ultrasound range. A soundwave is propagated in a manner in which energy is transmitted by molecules adjacent to each other in the medium, and it exhibits unique characteristics such as frequency and acoustic pressure according to the type of sound [23,24,25,26]. The intensity of sound decreases due to resonance and absorption as the propagation progresses, which is called attenuation. The main causes of sound attenuation can be largely divided into three factors, such the viscosity of the medium, heat conduction, and energy exchange between molecules. All attenuation is proportional to the square of the frequency, and the attenuation rate increases rapidly as the frequency increases [27].

If the wavelength of the target soundwave is sufficiently long compared to the size of the acoustic element, physical analysis within the acoustic element can be simplified regardless of the shape of the acoustic element. This is called “long wavelength approximation” and the acoustic elements are called lumped acoustic elements. The Helmholtz resonator, which is a representative example of such an acoustic element, is composed of a neck part comprising an open hole and a cavity part in the lower section. When soundwaves are transmitted from the outside to the Helmholtz resonator, the air in the neck is moved to the cavity by the soundwave, the air in the cavity is compressed, and the pressure in the cavity is increased. After this, the air is pushed out by the increased internal pressure, and the air is compressed again by external soundwaves. In this way, the air in the neck region vibrates up and down repeatedly, and the air in the cavity region repeatedly compresses and expands. The air motion of the neck and the air motion of the cavity can be interpreted as a mass and a spring, respectively. Using this, the Helmholtz resonator can be represented as a spring–mass system and analyzed mechanically. Like typical spring–mass systems, the Helmholtz resonator has a specific resonant frequency [23,28]. When a soundwave with the same frequency as the resonant frequency is applied to the Helmholtz resonator, a large radiation wave is generated through the resonance, causing a cancellation reaction with the external soundwave, and an acoustic attenuation effect appears. In the metamaterial field, acoustic attenuation by resonance could be considered to cause the negative modulus or density [5,25,26]. By using this property of the Helmholtz resonator, some research groups have introduced materials with negative modulus [19]. Moreover, using the fabrication method, some research groups have reported a microscale Helmholtz resonator based on the MEMS process [5,6,28,29,30]. Like this, the Helmholtz resonator with novel acoustic properties could be interesting material in many research fields. To utilize the resonant phenomenon of the Helmholtz resonator, as mentioned earlier, a Helmholtz resonator must satisfy the “long wavelength approximation”, and a Helmholtz resonator of such a small size is required to produce a Helmholtz resonator that attenuates high-frequency sounds. For example, a Helmholtz resonator that attenuates sound in an ultrasonic band of 20 kHz or higher frequency must be manufactured in a microscale. In the previous study, the fabrication of a Helmholtz resonator inspired sound absorbing material using MEMS technology was presented and an admirable acoustic attenuation result was observed [28]. In this study, based on the previous research, a Helmholtz resonator inspired acoustic element with various materials was fabricated by using MEMS manufacturing technology. Furthermore, acoustic attenuation characteristics were measured using various materials and micro-geometries, and analysis according to the elastic modulus of the materials was conducted. To increase usage of the acoustic element at broadband width, a sample with four sub-cavities was fabricated and an admirable attenuation result was observed.

## 2. Materials and Methods

### 2.1. Fabrication of Master Mold

To fabricate a master mold for the acoustic element composed of a micro-hierarchical structure inspired by the Helmholtz resonator, conventional photolithography was carried out twice. In the first process, the cavity part was made on substrate via the 1st photolithography without development process. The neck part was formed on the cavity-defined surface with precise alignment of the second photo mask followed by the 2nd photolithography. At this time, SU-8 series (Microchem) was used as photoresist (PR). After the 2 photolithography processes, through the development process and rinsing by using deionized water, the fabrication process for the master mold was successfully completed.

### 2.2. Fabrication of the Acoustic Element (Soft Lithography)

The acoustic element was fabricated by soft lithography with polydimethylsiloxane (PDMS), a thermosetting polymer with low Young’s modulus (~1.8 MPa). Generally, PDMS is utilized in a 10:1 mixture ratio of the elastomer base and the curing agent (Sylgard 184, Dow Corning Corp, Midland, TX, USA). To achieve uniform thickness among experimental samples, a constant amount of PDMS mixture was poured on the master mold. After the degassing process in vacuum chamber, the PDMS mixture was cured in oven at 70 °C for 2 h. The fully cured PDMS was carefully demolded from the master mold. Through this process, the acoustic element inspired by Helmholtz resonator was prepared.

### 2.3. Fabrication of the Acoustic Element (Rigiflex Lithography)

The acoustic element composed with UV-curable material was fabricated by using “rigiflex (rigid + flexible)” lithography, rigid enough to replicate fine structures up to a few nanometers but also flexible on the film support. A polyurethaneacrylate (PUA) (product name: MINS-301 RM, MINS-311 RM, Korea) was used as resin of rigiflex lithography and the PUA was cured for 3 min in a UV chamber with 10 mW cm^−2^ UV intensity after an adequate amount of PUA resin was dropped onto the mold. The acoustic element consisted of the PUA prepared with careful demolding.

### 2.4. Measurement

The acoustic element composed with a microscale hierarchical structure was optically characterized by optical microscopy (LV150L, Nikon, Tokyo, Japan) and scanning electron microscopy (SEM) (S-4800, Hitachi, Marunouchi, Japan) after Pt sputtering to form a thin metal layer (<5 nm) to prevent electron charging.

### 2.5. Acoustic Attenuation Experiment

To observe the acoustic attenuation performance, the experimental sample was located in the center of the transducers (transmitter and receiver) and sound pressure change between the transmitter and the receiver in a water bath was measured. The water bath could be considered a reciprocal environment since the dimension of the water bath was sufficiently large compared to the target acoustic frequency and measured sound pressure at the receiver represented the sound pressure level.

## 3. Results and Discussion

**Figure 1:**
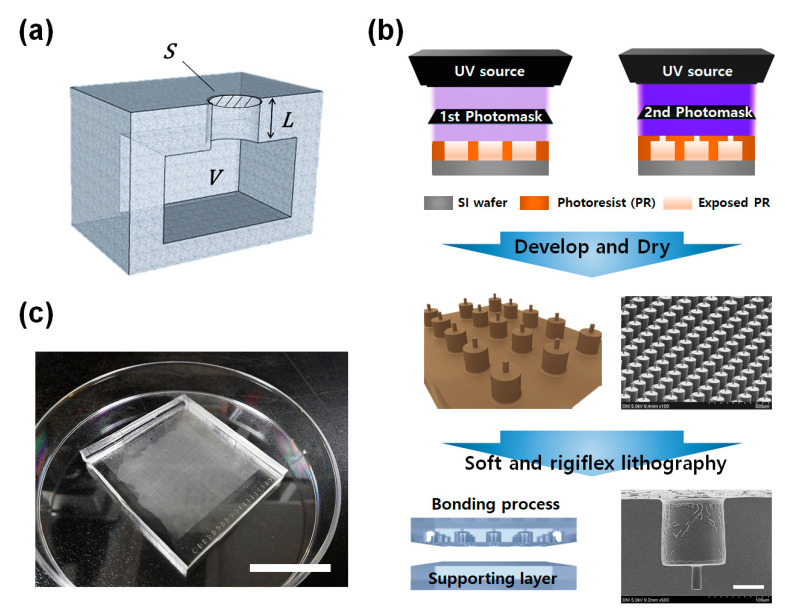
(**a**) Illustration of the Helmholtz resonator. (**b**) Fabrication process of samples. (**c**) Photograph of sample. Scale bar is 200 and 50 μm, respectively.

As shown in Figure 1a, the Helmholtz resonator is composed of a neck part composed of an open hole and a cavity part at the lower part. The resonant frequency of a conventional Helmholtz resonator composed of a cavity of volume *V* and a neck of area *S* and length *L* is expressed by

$$2\pi f=c\;\times \;\sqrt{\frac{S}{L\prime V}}$$

where *c* is the speed of sound in a particular medium, *L*’ is the effective length of the neck with a radius a due to the influence of the air outside and inside the Helmholtz resonator. Effective length, *L*’, is expressed as *L*’ = *L* + 1.7*a* (outer end flanged) and *L*’ = *L* + 1.4*a* (outer end unflanged) according to the shape of the Helmholtz resonator.

As shown in Figure 1b, a two-step photolithography process for fabrication of the master mold was performed to fabricate the shape of the cavity and neck to create the microscale acoustic element whose shape resembles the Helmholtz resonator. Then, softlithography and rigiflex lithography using PDMS (polydimethysiloxane) and the PUA were conducted to replicate the micro-structures from the master mold. Since the attenuation experiment was implemented in the water bath, to prevent the filling of external water into the micro-structures and maintain an air layer in the sample, the sample was attached with a supporting layer. Finally, the shape of the microscale Helmholtz resonator-like structures was well realized, as shown in the SEM image (Figure 1c).

**Figure 2:**
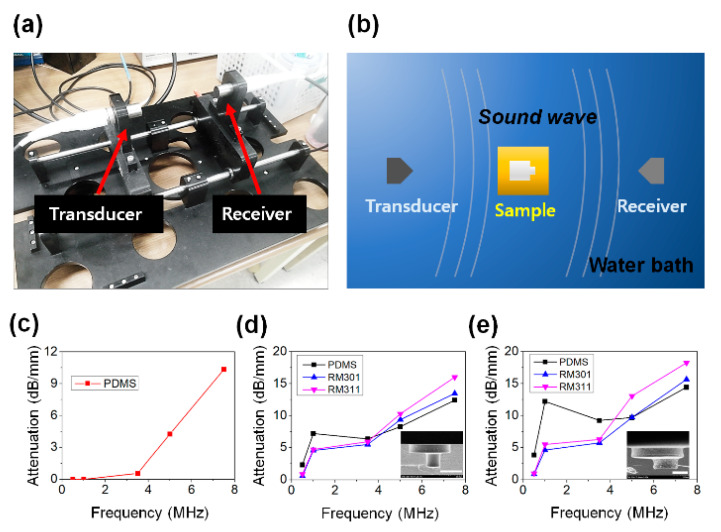
(**a**) Photograph of the experimental jig. (**b**) Illustration of the experimental setup. (**c**) Attenuation result of non-patterned polydimethylsiloxane(PDMS). (**d**) Attenuation result of 60 μm diameter sample with various materials. (**e**) Attenuation result of 40 μm diameter sample with various materials.

As shown in Figure 2a,b, to measure the acoustic attenuation performance, the experimental system was set with the acoustic elements which had around 47,000 hierarchical micro-structures arranged in an area of 40 × 40 mm. Although the acoustic element inspired by the Helmholtz resonator did not follow the above equation exactly, considering that the material affects not only the Helmholtz resonator inspired structures, resonance frequency could be predicted as ultrasonic range based on “long wavelength approximation” since all dimensions of samples were under 100 µm. A fabricated sample was placed in the center of the experimental jig and a soundwave was generated using a transducer. The acoustic signal passing through the sample was measured by the receiver to analyze the degree of acoustic attenuation.

The microscale structured acoustic elements have a resonant frequency in the MHz range. Since soundwaves in the MHz range easily dissipate in the air, the experiment was conducted in a water bath, and the spacing of the measuring element was adjusted in consideration of the planar wave (Figure 2b). Since the attenuation of soundwaves is proportional to the square of the frequency, the attenuation increases as the frequency increases. First, to find out the attenuation effect of the material itself, a measurement was conducted using a PDMS slab without any pattern. As shown in Figure 2c, no specific attenuation characteristics were observed in the non-patterned PDMS slab. When a soundwave of a resonant frequency is applied to the Helmholtz resonator, the soundwave of the opposite phase emitted from the Helmholtz resonator causes destructive interference with the input soundwave, resulting in a sound absorption effect at a specific frequency. In this study, observing the attenuation change according to material is important since a lot of micro-resonators (around 47,000) were reacted by the sound pressure at the same time, causing not only the vibration effect of the medium in the resonator but also the vibration of the material effect of the resonator. In order to examine the effect of the material, an experiment was conducted using various materials such as PDMS and UV-curable materials which include hard-type polyurethaneacrylate (PUA) and soft-type PUA. The hard PUA, which is called RM311, has 300 MPa of elastic modulus and soft PUA (RM301) has 150 MPa. As a result of conducting a test using a sample with a cavity diameter of 60 μm, the acoustic element made of PDMS showed a large attenuation characteristic around 1 MHz (Figure 2d). The attenuation rate at 1 MHz was measured as 7.20, 4.71, and 4.56 dB mm^−1^ for the acoustic elements composed of PDMS, hard PUA, and soft PUA, respectively. As shown in Figure 2e, a similar trend was also observed in the experimental results using a sample with a cavity diameter of 40 μm. At 1 MHz, the attenuation rates of the acoustic elements composed of PDMS, RM311, and RM301 were measured to be 12.21, 5.48, and 4.61 dB mm^−1^, respectively.

Specifically, the acoustic element composed of PDMS showed a maximum attenuation rate of 7.60 dB mm^−1^ higher than that of other materials. This acoustic attenuation phenomenon occurs because a material with a low elastic modulus could radiate a relatively large number of reflected acoustic signals that can cause resonance while actively deforming its shape against sound pressure. In other words, the fabricated acoustic element generates large radiated sound pressure by the resonance and has an acoustic attenuation effect.

**Figure 3:**
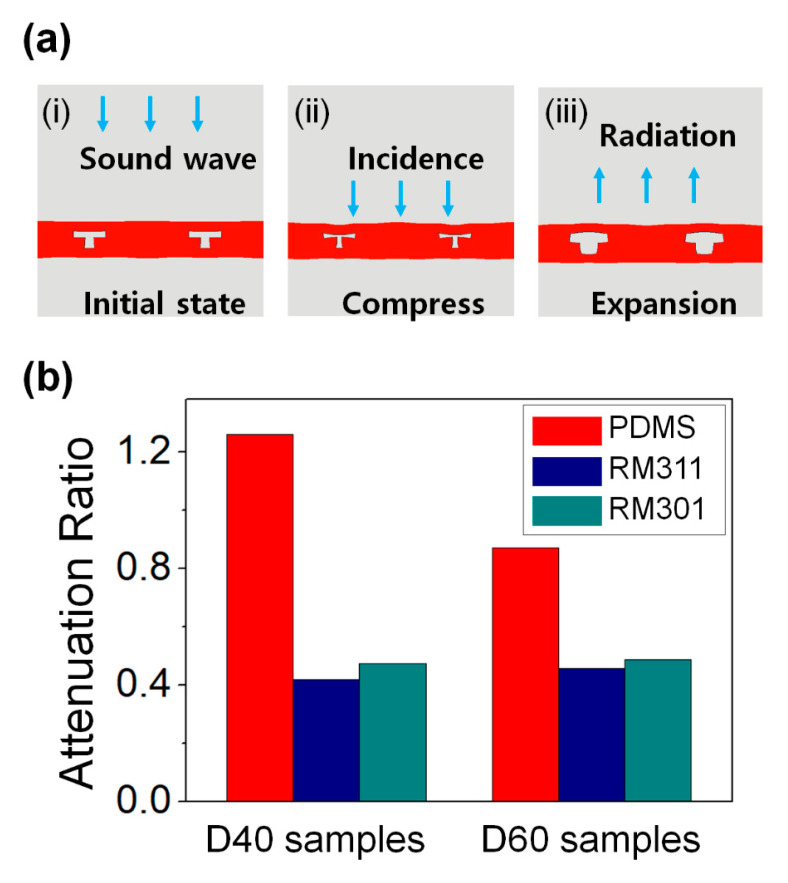
(**a**) Simulation result of acoustic attenuation by sample. (**b**) Attenuation ratio according to various materials.

The acoustic attenuation performance of the fabricated acoustic element using various materials was observed. However, unlike an acoustic element composed of soft PDMS, a special attenuation performance did not appear in an acoustic element composed of a relatively hard UV-curable material. As shown in Figure 3a, in the acoustic attenuation mechanism simulation, (i) when a sound wave is applied to a micro-scale acoustic element, (ii) the element contracts by the sound wave, and (iii) radiation by expansion occurs, resulting in an acoustic attenuation effect. The 1.8 MPa as Young’s modulus of material and 1 MHz soundwave were used as initial conditions in simulation. Additionally, the planar wave was incident to the acoustic element and all elements were considered reciprocal and isotropic. PDMS is a silicone-based rubber and is used in various fields due to its low surface energy, high transparency, and high replication performance. The Young’s modulus of PDMS is 1.8 MPa, and its shape can be easily changed according to external stimuli. However, as shown in Table 1, since RM311 and RM301 have a relatively large Young’s modulus of 300 and 150 MPa, respectively, it is difficult to realize sound attenuation with a mechanism such as Figure 3a. To observe the attenuation performance intuitively, by using the ratio of the attenuation rate at 1 MHz and the attenuation rate at 5 MHz, attenuation result according to materials is more clearly indicated (Figure 3b). Through the attenuation ratio between 1 and 5 MHz, attenuation performance could be shown easily. Generally, the attenuation ratio is much less than 1 since the attenuation rate at 5 MHz must be higher than the attenuation rate at 1 MHz. For example, in the non-patterned PDMS case (Figure 2c), the attenuation ratio value was just 0.24. If the sample exhibits good attenuation rate by resonance at 1 MHz, the attenuation ratio would be close to 1. Thus, if acoustic attenuation by resonance occurs, the attenuation ratio would be increased. Therefore, the attenuation ratio means the larger the attenuation ratio, the greater the attenuation. When the diameter of the cavity was 40 μm, the sample made with PDMS showed a high attenuation ratio of 1.26, but the samples made with RM311 and RM301 showed a low ratio of 0.42 and 0.47, respectively. Moreover, when the diameter of the cavity was 60 μm, the sample made with PDMS showed an attenuation ratio of 0.87, whereas the samples made with RM311 and RM301 showed values of 0.46 and 0.49, respectively. In both cases, as in the simulation results, the sample composed of PDMS showed high attenuation performance. Moreover, the attenuation ratio of the RM311 with a higher Young’s modulus was observed to be lower than the attenuation ratio of the RM301 and this could be additional evidence of the simulation result.

**Figure 4:**
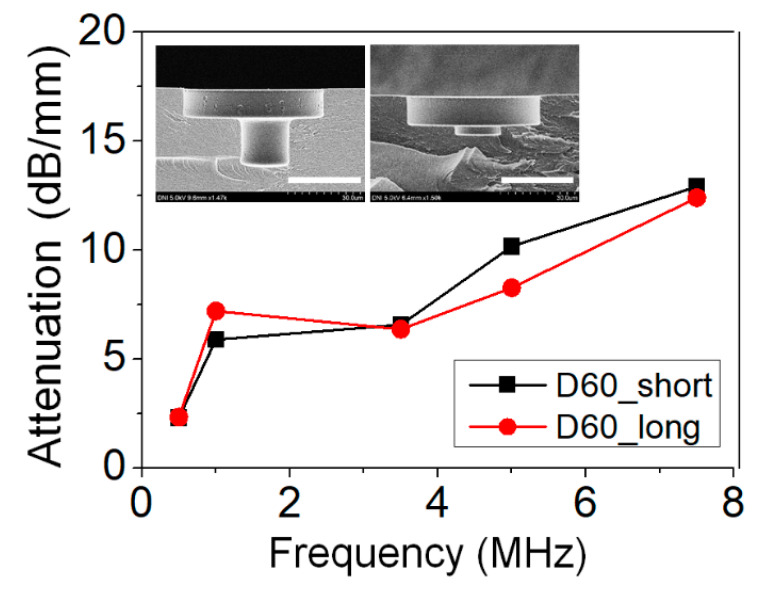
Attenuation result depending on height of the neck. Scale bar is 30 μm.

In addition, in order to find out the difference in acoustic attenuation performance according to the size of the neck, a sample with a cavity volume of 60 μm in diameter and a different height of the neck was made in order to perform acoustic attenuation experiments. The attenuation test was conducted using samples with a neck length of 11.5 μm and a sample of 3.6 μm, whose SEM images are shown in Figure 4. As a result, the acoustic attenuation characteristics were very poor in the case of the sample with the short neck length. This is because the air in the neck and the cavity interacts and compresses and expands. When the acoustic element contracts by soundwaves, the air pressure in the neck and cavity changes instantaneously due to the difference in the cross-sectional area of the neck and cavity of the acoustic element, and contraction occurs, and accordingly, radiation occurs with a greater pressure. However, when the length of the neck is short, there is not enough air in the neck for this mechanism to occur, and the acoustic attenuation rate decreases. Moreover, this is more clearly exhibited by using the attenuation ratio. When expressed as the ratio of the attenuation rate at 1 MHz and the attenuation rate at 5 MHz, the attenuation ratio of the long neck and the short case was 0.87 and 0.58, respectively. In samples with a neck length of 11.5 μm, the attenuation ratio was shown to be 1.5 times higher than the sample of the 3.6 μm case. Through this experiment, it was found that the length of the neck has an important effect on the acoustic attenuation performance.

**Figure 5:**
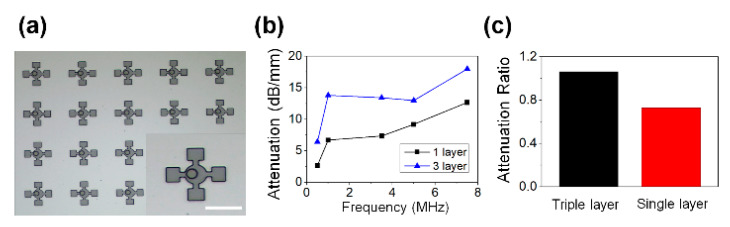
Microscopic image of sample with sub-cavities. Scale bar is 100 μm. (**b**) Attenuation result of samples. (**c**) Attenuation ratio data of triple-layer sample and single-layer sample.

Finally, to observe the performance of the acoustic element for broadband width, an acoustic element in a shape in which micro-resonators of various sizes coexist was fabricated, as shown in Figure 5a. A sample with four additional cavities of 40 × 40 μm in the neck of 20 μm in diameter and a cavity with a diameter of 60 μm was prepared. Since each cavity and neck in the unit acoustic element could play the role of “spring–mass system”, to induce the resonance of the wide range, the designed acoustic element for broadband width was fabricated with an additional four springs and masses in the unit acoustic element. As shown in Figure 5b, the attenuation value was not relatively high in the single layer sample, but it was confirmed that the sample in which three samples were stacked had sufficiently high attenuation performance to compensate for this. The attenuation performance of the single sample and stacked sample at 1 MHz was 6.70 and 13.81 dB mm^−1^, respectively, and the attenuation ratio shown in Figure 5c also showed high attenuation performance of 0.73 and 1.06. In particular, in the case of the three-layer stacked sample, only the attenuation rates of 1 and 3.5 MHz were shown to be higher than the attenuation rate at 5 MHz in this experiment. This result was meaningful in that acoustic attenuation characteristics could be exhibited for a wide range of 1 to 3.5 MHz, and this means that this acoustic element could be utilized at the broadband width. Through this unique design with additional cavity and neck, the possibility for a new design for the broadband width sample could be suggested.

## 4. Conclusions

In this study, a micro-structured acoustic element inspired by the Helmholtz resonator applied with “long wavelength approximation” was fabricated by using conventional photolithography and soft lithography and rigiflex lithography. The acoustic element with hierarchical micro-structure arrays was composed of polymers with various Young’s modulus values (1.8~300 MPa) and could attenuate soundwaves via acoustic resonance. Through various experiments in a water bath, the novel acoustic attenuation characteristics at ultrasonic range were verified. In addition, the mechanism of acoustic attenuation characteristics was analyzed according to the shape of micro-structure in the acoustic element and various materials based on the Young’s modulus. Through the experiment with various materials, change in the acoustic attenuation was observed despite the samples having the same dimensions. High acoustic attenuation performance of up to 13 dB mm^−1^ was observed and the possibility of utilization with broadband width was confirmed. Based on the admirable performance observed in this study, we expect that we could provide new perspectives and topics about microscale acoustic elements composed of various materials to readers and believe that this acoustic element could be utilized in various fields, such as medical, military, industrial fields, and so on.

## Figures and Tables

**Table 1:** Young’s modulus of materials.

	PDMS	RM311	RM301
Young’s modulus (MPa)	1.8	300	150
